# Development of novel combinations of targeted and immunotherapies by understanding immune resistance using a high throughput assay of T cell mediated cytotoxicity

**DOI:** 10.1186/2051-1426-1-S1-P164

**Published:** 2013-11-07

**Authors:** Shruti Malu, Rina Mbofung, Jahan Khalili, Nikunj Satani, Marie-Andrée Forget, Cara Haymaker, Florian Muller, Chantale Bernatchez, Laszlo Radvanyi, Patrick Hwu

**Affiliations:** 1 Department of Melanoma Medical Oncology, University of Texas MD Anderson Cancer Center, Houston, TX, USA; 2 Department of Genomic Medicine, University of Texas MD Anderson Cancer Center, Houston, TX, USA

## 

The ability of the immune system to treat established tumors is very well recognized. Immunotherapeutic approaches such as Adoptive T cell therapy and anti-CTLA-4 antibody treatment can result in long-term responses in some patients with metastatic melanoma. However, the majority of patients do not respond. A thorough understanding of the mechanisms by which tumors resist T cell mediated killing is thus crucial for the design of appropriate combinations of immune- and targeted therapies and for defining potential biomarkers that can distinguish responders, with the ultimate goal of achieving durable disease free regressions in many more patients. To approach these two different but overlapping goals, we adapted an in vitro assay of T cell mediated cytotoxicity for high throughput screening. In this assay, cytotoxicity is measured by intracellular staining of active Caspase-3 in tumor cells followed by flow cytometry in 96 well plates. A number of human melanoma cell lines and their autologous TILs (Tumor infiltrating lymphocytes) were tested and candidate pairs were chosen for a screen of 850 compounds to identify drugs that are synergistic with T cell mediated killing and hence can be used in combinations with immunotherapies. In this study, we are focusing on a class of drugs that inhibit Heat shock protein 90 (Hsp90). Briefly, the tumor line is treated with the drugs for 24 hours, followed by addition of autologous T cells that kill these treated tumor cells. A combo score for apoptosis in tumor cells is calculated for each drug. A higher score indicates that the treatment of the tumor with the drug renders it more sensitive to T cell mediated killing. We show that three Hsp90 inhibitors, 17 DMAG, 17AAG and BIIB021, show a high combo score with T cell mediated cytotoxicity. The synergy of this drug treatment with T cell mediated cytotoxicity suggests that this category of compounds maybe beneficial in combination therapies. We are currently testing 17AAG with immunotherapies such as anti CTLA-4, anti PDL1 and anti OX40 in different tumor models in mice. Experiments are underway to understand the changes in signaling pathways (by RPPA) and gene expression (by microarray) on treatment of different tumor lines with 17AAG to mechanistically understand the role of Hsp90 in development of resistance to direct T cell mediated cytotoxicity. This screen will serve as a pipeline for combinations that would lead to new clinical trials as well as identify novel mechanisms of cancer immune resistance.

